# Molecular mechanisms of Mmd2 gene in regulating growth of the Pacific white shrimp *Litopenaeus vannamei*

**DOI:** 10.1007/s42995-024-00273-7

**Published:** 2025-02-15

**Authors:** Shuqing Si, Xiaojun Zhang, Yang Yu, Xiaoyun Zhong, Xiaoxi Zhang, Jianbo Yuan, Ka Hou Chu, Fuhua Li

**Affiliations:** 1 CAS and Shandong Province Key Laboratory of Experimental Marine Biology, Center for Ocean Mega-Science, Institute of Oceanology, Chinese Academy of Sciences, Qingdao, 266071 China; 2 Laboratory for Marine Biology and Biotechnology, Qingdao Marine Science and Technology Center, Qingdao, 266237 China; 3 College of Earth Science, University of Chinese Academy of Sciences, Beijing, 100049 China; 4 Key Laboratory of Breeding Biotechnology and Sustainable Aquaculture, Chinese Academy of Sciences, Wuhan, 430072 China; 5 Southern Marine Science and Engineering Guangdong Laboratory (Guangzhou), Guangzhou, 510301 China; 6 School of Life Sciences, The Chinese University of Hong Kong, Shatin, NT, Hong Kong China

**Keywords:** Pacific white shrimp, Monocyte to macrophage differentiation factor 2, Growth-inhibiting gene, Function, Ras signaling pathway

## Abstract

Growth of the Pacific white shrimp *Litopenaeus vannamei*, the most important farmed crustacean, has consistently been a focal point for breeders. Over the past decades, some candidate genes for shrimp growth have been identified. However, further research is needed to elucidate the molecular regulatory mechanism of these genes. *LvMmd2* was previously identified as a candidate gene that may inhibit the growth of *L. vannamei*. In this study, we analyzed the genotype and expression of the *LvMmd2* gene in a breeding family and indicated its role as a growth-inhibiting gene. We found that LvMmd2 co-localized with its homolog LvPAQR3 at the Golgi apparatus. Using co-immunoprecipitation (Co-IP) and DUAL membrane system yeast two-hybrid (MbY2H), we indicated the interactions between LvMmd2 and LvPAQR3, LvPAQR3 and LvRaf1, as well as LvMmd2 and LvRho. These results suggest that *LvMmd2* directly and indirectly regulates the Ras signaling pathway. Furthermore, we show that the *LvMmd2* gene may indirectly affect the PI3K/AKT, insulin, and Hippo signaling pathways to regulate cell proliferation and differentiation via LvPAQR3 and LvRaf1. Through transcriptome and MbY2H analyses, we have also revealed the interaction between LvMmd2 and proteins involved in growth, immunity, protein transport, synthesis, and modification. These findings demonstrate the various molecular pathways through which *LvMmd2* regulates *L. vannamei* growth. This study provides insights into the mechanism of shrimp growth regulated by *Mmd2*, enhances our understanding of *LvMmd2* function, and highlights its potential application in shrimp breeding.

**Supplementary Information:**

The online version contains supplementary material available at 10.1007/s42995-024-00273-7.

## Introduction

Penaeid shrimp are a major target group in commercial fisheries and aquaculture (Cheng et al. 2018; De Grave and Fransen 2011). In recent decades, numerous efforts have been made to improve the important economic traits of shrimp, such as growth, fertility, disease resistance, and stress tolerance (Yuan et al. 2023). Of these traits, growth has been the focus of breeders as it is directly related to shrimp production. However, the relatively weak research base and lack of suitable model organisms have hindered the progress of research on growth-related genes in shrimp, resulting in a limited understanding of the molecular mechanisms of growth in these species.

The growth of shrimp is polygenic controlled by multiple quantitative trait loci (QTLs) and genes (Wang et al. 2020). In earlier studies, some growth-related genes have been identified and investigated in shrimp, such as *myostatin* (Lee and McPherron 2001; Lee et al. 2011; Yan et al. 2020), *myosin* (Kamimura et al. 2008), *FABP* (fatty-acid-binding protein) (Storch and Thumser 2010), and *amylase* (Warren et al. 2011). With the completion of a high-density genetic linkage map and a whole genome map of the Pacific white shrimp *Litopenaeus vannamei* (Yu et al. 2015; Zhang et al. 2019), the capacity to accurately locate genes associated with shrimp traits using QTL and genome-wide association studies (GWAS) has improved. Recently, researchers have identified multiple genes related to growth in *L. vannamei*. These genes include *PKC-δ* (protein kinase C-delta) and *Rap-2a* (Ras-related protein) (Yu et al. 2019), *SRC* (class C scavenger receptor) (Wang et al. 2019), *Mmd2* (monocyte to macrophage differentiation factor 2) (Wang et al. 2020), *dCMPD* (deoxycytidylate deaminase), and *NPTK* (non-receptor protein tyrosine kinase) (Lyu et al. 2021). Among these studies, Wang et al. (2020) found that the SNP associated with the growth of *L. vannamei* located on the *Mmd2* gene, with a nonsynonymous mutation named MMD_5 contributing the most to the trait, explaining ~ 10% of the phenotypic variance. This finding suggests that *Mmd2* may serve as a crucial candidate gene for shrimp growth (Wang et al. 2020).

The Mmd2 gene encodes the PAQR10 protein, which belongs to the progesterone and adiponectin receptor (PAQR) family (Tang et al. 2005; Thomas et al. 2007). PAQR is a group of membrane receptor proteins that share a conserved seven-transmembrane domain. In prokaryotes, PAQR family genes encode hemolysin-type proteins. In eukaryotes, these genes code for adiponectin or progestin-related receptors and have extensive and apparent ligand specificity (Baida and Kuzmin 1995, 1996; Tang et al. 2005). The adiponectin receptors (PAQR1 and PAQR2) were the first family discovered, and they play a crucial role in regulating glucose and lipid metabolism (Yamauchi et al. 2001, 2002, 2003). PAQR3, renamed Raf1 kinase trapping to Golgi (RKTG), was found to sequester Raf1 kinase to the Golgi apparatus, inhibiting the Ras-Raf-ERK signaling pathway (Feng et al. 2007). The Raf1 protein kinase is an important component of the Ras/Raf/MEK/ERK signaling pathway in mammals, and its knockdown in mice could lead to cell apoptosis, mainly in the liver and hematopoietic system (Chen et al. 2001; Hüser et al. 2001). In addition, there is increasing evidence that Raf1 may use multiple effectors, including its established target MEK, to mediate cellular functions. PAQR3 negatively regulates the PI3K/AKT signaling pathway by interacting with the catalytic subunit of PI3K (p110a), and subsequently negatively regulates the insulin signaling pathway (Qiao et al. 2021; Xiao et al. 2022). PAQR10 and PAQR11, which have the highest similarity to bacterial hemolysin proteins, are most likely the descendants of the original PAQR genes (Jin et al. 2012a). However, there is limited research on PAQR10 (Mmd2). Researchers have found that PAQR10 and PAQR11 are involved in delaying and prolonging Ras signaling, as well as regulating the Ras pathway, which plays a crucial role in normal cell proliferation and serves as a nodal point for multiple growth signaling pathways (Cass and Meinkoth 1998; Crespo and Leon 2000; Jin et al. 2012a, b, 2015). Considering the potentially important role of PAQR10 in growth and development, further research is needed to elucidate the molecular mechanisms of the Mmd2 gene in regulating shrimp growth.

Recently, we cloned and identified the *Mmd2* gene of *L. vannamei* (*LvMmd2*) and found that body length and weight gain could be significantly enhanced following the knockdown of *LvMmd2* by RNA interference (RNAi), suggesting its role as an inhibitor of shrimp growth (Si et al. 2023). However, the mechanism through which *LvMmd2* exerts negative regulation on shrimp growth remains unclear. In this study, we first support the genotype of *LvMmd2* in a shrimp family and indicate its role as a growth suppressor gene. Subsequently, utilizing evidence from cellular localization, co-immunoprecipitation (Co-IP), and DUAL membrane system yeast two-hybrid assay (MbY2H), along with transcriptome analysis after RNAi, we demonstrated that *LvMmd2* regulates shrimp growth through various signaling pathways, including Ras, Hippo, and insulin signaling pathways. These results provide valuable insights for future research and use of the Mmd2 gene in shrimp and crustaceans.

## Materials and methods

### Experimental animals

For the RNA interference (RNAi) experiment, ~ 1000 *L. vannamei* were obtained from Rizhao Xingguang Marine Ranch Fishery Co (Rizhao, Shandong, China) and shipped to the Institute of Oceanology, Chinese Academy of Sciences (Qingdao, Shandong, China). These shrimp came from the same culture family and had similar body length and weight. Cultures were maintained at 25 °C and natural seawater (30 PSU). The seawater was changed every day. Shrimp were fed food pellets (Dell Feed Company, Yantai, China) three times a day, at 0.016 g of food per g of shrimp body mass. A total of 480 individuals were used for the RNA interference (RNAi) experiment; these had an average initial body weight of 3.5 ± 1.8 (SD) g and an average initial body length of 6.5 ± 1.6 (SD) cm.

For the genotype and gene expression analysis experiment, another 100 shrimp were selected from the same family (Family 21253), which were bred in Bohai Aquaculture Breeding (Hainan) Co. Ltd (Wenchang, Hainan, China). These shrimp have been cultured for a uniform duration of 30 days; however, they exhibit variations in size. They were sorted by weight, with 20 individuals from the top 30 were selected as the fast-growing group (3.3 ± 1.1 g), and 20 individuals from the bottom 30 were selected as the slow-growing group (1.2 ± 0.5 g).

### Preparation of double-strand RNA (dsRNA) and RNA interference

To knock down the target gene *LvMmd2*, two pairs of primers, EGFP-F/R and LvMmd2-F/R, were designed to amplify the *EGFP* and *LvMmd2* genes. Primers for dsEGFP-F/R and dsMmd2-F/R, with T7 promoter sequences, were used to clone a 289 bp DNA fragment and a 507 bp DNA fragment for dsRNA synthesis. PCR was performed according to the manufacturer’s protocol of the Premix Ex Taq™ Hot Start Version (TaKaRa, Japan). PCR products were detected by electrophoresis on 1% agarose gel and purified using the MiniBEST DNA fragment purification kit (TaKaRa, Japan). The purified products were used to synthesize the corresponding dsRNAs using the Transcript Aid T7 High Yield Transcription Kit (Thermo Fisher Scientific, USA). The dsRNAs were then purified using a phenol and chloroform mixture, and any redundant single-strand RNA was removed by RNaseA. The qualified dsRNAs were stored at − 80 °C for subsequent RNA interference experiments.

Three doses of dsRNA (2, 6, 10 μg) were injected into the last abdominal segment of each shrimp. The same doses of EGFP dsRNA and 10μl PBS were injected into the shrimp as the control group. After detecting the transcription level of *LvMmd2* at 48 h post injection, the dosage of 2 μg dsRNA was selected for further RNAi experiments. A total of 480 shrimp (premolt stage D1–D2) were randomly divided into three groups: the dsMmd2 experimental group and two control groups (dsEGFP and PBS). Each group consisted of 160 individuals, which were further divided into three replicates immediately and raised in separate tanks. 2 μg of dsMmd2, 2 μg of dsEGFP, and 10 μl PBS were injected into the last abdominal segment of each group of shrimp. The same injection was repeated every four days, and the experiment lasted for two weeks (Si et al. 2023). Before and after the experiment, the body weight and body length of every shrimp were measured and recorded.

### Tissue collection

For the RNA interference (RNAi) experiment, the knockdown efficiency of *LvMmd2* was detected after 48 h of injection. To reduce the inter-individual error of shrimp, the hepatopancreas of four shrimp was taken as one biological replicate. Sixteen shrimp from each separate tank were divided into four biological replicates, and a total of twelve biological replicates were obtained from three separate tanks with the same group (treatment group or control groups). The muscle was collected in the same manner. For the dsMmd2 group (and its control), three hepatopancreas replicates were collected for transcriptome sequencing (OE Biotech Co., Ltd., Shanghai, China).

For the genotype and gene expression analysis experiment, muscle and hepatopancreas samples were individually collected from 40 individuals of Family 21253, and the tissues of each individual were stored separately. All tissues were frozen in liquid nitrogen and stored at − 80 °C before analysis.

### DNA extraction

DNA was extracted from the muscles using the plant genome DNA kit (TIANGEN, Beijing, China), following the manufacturer’s instructions. The purity and quality of the DNA extracts were determined using a NanoDrop 2000 spectrophotometer (Thermo Fisher Scientific, USA) and 1% agarose gel electrophoresis. The high-quality DNA samples were stored at − 20 °C before analysis.

### RNA isolation and cDNA synthesis

RNA was isolated from the different tissues of the shrimp using the RNAiso Plus reagent (TaKaRa, Japan), following the manufacturer’s instructions. Approximately 100 mg of tissue from each sample was used for RNA extraction. The quality and concentration of RNA were assessed by electrophoresis on a 1% agarose gel and Nanodrop 2000 (Thermo Fisher Scientific, USA). All cDNA samples were synthesized from 1 µg total RNA with the PrimeScript RT Reagent Kit (TaKaRa, Japan). According to the manufacturer’s instructions, genomic DNA (gDNA) was removed using a genomic DNA eraser buffer, followed by the synthesis of the first strand cDNA using PrimeScript RT Enzyme with random primers. Finally, all cDNA samples were stored at − 80 °C before analysis.

### Quantitative real-time PCR analysis (qRT-PCR)

The SuperReal PreMix Plus (SYBR Green) (Tiangen, China) was used to detect the expression of related genes in RNAi-treated samples, with 18S rRNA selected as an internal reference gene (primer sequences are listed in Supplementary Table S1). QRT-PCR was performed using the Eppendorf Mastercycler ep realplex (Eppendorf, Hamburg, Germany). All reagents were mixed according to the manufacturer’s protocol, and each biological replicates included four technical replicates. The PCR steps were as follows: 94 °C for 2 min, 40 cycles of 94 °C for 15 s, annealing temperature for 20 s, and 72 °C for 20 s. The specificity of the primers was tested using melting curves. Finally, the relative expression level of the genes was calculated using the 2^−△△Ct^ method (Livak and Schmittgen 2001).

The data obtained from each qRT-PCR experiment were analyzed to calculate the mean and standard deviation of triplicate assays. Statistical significance between the control and different treatment groups was determined by one-way ANOVA using SPSS (https://www.ibm.com/cn-zh/analytics/spss-statistics-software) (version 20), with Prism software used to visualize column plots.

### Gene cloning and sequence analysis

To obtain the full length of *LvMmd2* and other genes, PrimeStar GXL DNA polymerase (TaKaRa, Japan) was used for PCR amplification (for primer sequences see Supplementary Table S2). The PCR profile was as follows: 1 cycle of denaturation at 98 °C for 5 min, 35 cycles of amplification (98 °C for 10 s, annealing temperature for 15 s, 68 °C for extension), and finally extension at 68 °C for 10 min. The PCR products were qualified using 1% agarose gel electrophoresis, collected and purified using the Gel Extraction Kit (Omega, Norcross, GA, USA). The purified products were then inserted into the pMD19-T vector (TaKaRa, Kyoto, Japan), and transformed into Trans5α chemically competent cells (TransGen Biotech, Beijing, China) for sequencing.

We used ORF Finder (https://www.ncbi.nlm.nih.gov/orffinder/) and ExPASy translation tool (http://web.expasy.org/translate/) to obtain the deduced amino acid sequences. The amino acid sequences were further analyzed by SMART (http://smart.embl-heidelberg.de/). In addition, we used STRING (https://cn.string-db.org/) to predict the genes interacting with *LvMmd2*.

### Plasmid construction

Construction of co-immunoprecipitation (Co-IP) expression plasmid: the pDHsp-70-V5-His vector and pDHsp-70-Flag-His vector were digested with the appropriate restriction enzymes (*Hin*d III and *Bam*H I) to generate linearized vectors. The complete ORF region of *LvMmd2* was amplified using the primers pDHsp-70-*LvMmd2*-F/R. The half region of *LvPAQR3* (*LvPAQR3*-ΔXb: with the partial transmembrane domain) was amplified using pDHsp-70-*LvPAQR3*-ΔXb-F/R. The C-terminus of *LvPAQR3* (*LvPAQR3*-ΔC) was amplified using pDHsp-70-*LvPAQR3*-ΔC-F/R. The STKC domain of *LvRaf1* was amplified using pDHsp-70-*LvRaf1*-ΔSTKC-F/R. The primers with homologous arms on both sides of the digested vector are listed in Supplementary Table S3. *LvMmd2* was inserted into the digested pDHsp-70-Flag-His vector using the In-Fusion^®^ Snap Assembly Master Mix (TaKaRa, Kyoto, Japan) to construct the Flag-His-tagged *LvMmd2* expression plasmid. *LvPAQR3*-ΔXb was inserted into the digested pDHsp-70-V5-His vector to construct the V5-His-tagged *LvPAQR3*-ΔXb expression plasmid, and *LvPAQR3*-ΔC was inserted into the digested pDHsp-70-V5-His vector to construct the V5-His-tagged *LvPAQR3*-ΔC expression plasmid. *LvRaf1*-ΔSTKC was inserted into the digested pDHsp-70-Flag-His vector to construct the Flag-His-tagged *LvRaf1*-ΔSTKC expression plasmid. *LvRho* was inserted into the digested pDHsp-70-V5-His vector to construct the V5-His-tagged *LvRho* expression plasmid.

The construction of cell localization plasmid: the pDHsp-70-V5-His vector and the pDHsp-70-Flag-His vector were digested with appropriate restriction enzymes (*Eco*R I and *Bam*H I) to generate linearized vectors. The primers pDhsp-70-EGFP-F/R and pDhsp-70-mCherry-F/R (Supplementary Table S3) were then used to amplify EGFP and mCherry tag protein fragments, respectively. The purified products were inserted into their corresponding linearization vectors using the in-fusion HD cloning kit (Clontech, Mountain View, CA, USA) to obtain plasmids pDhsp-70-EGFP-flag-His and pDhsp-70-mCherry-V5-His. The plasmids were then linearized using *Bam*H I and *Hin*d III, and the ORF regions of *LvMmd2* and the C-terminus of *LvPAQR3* were amplified using the primer pDhsp-70-*LvMmd2*-EGFP-F/R and pDhsp-70-*LvPAQR3*-ΔC-mCherry-F/R (Supplementary Table S3), respectively. The purified products were then inserted into the corresponding linearization vectors using the in-fusion HD cloning kit to obtain plasmids pDhsp-70-*LvMmd2*-EGFP-flag-His and pDhsp-70-*LvPAQR3*-ΔC-mCherry-V5-His.

All of the recombinant plasmids listed in Supplementary Table S4 were used to transform Trans5α chemically competent cells (TransGen Biotech, Beijing, China) for sequencing. The positive clones were used to inoculate 10 mL of low-salt LB medium for zeocin selection and culture. The plasmids were then extracted using the endotoxin-free plasmid Mini Kit I (Omega, Lyndhurst, NJ) and stored at − 20 °C until use.

### Cells culture and transient transfection

Sf9 insect cells were cultured in Sf-900™ II SFM (Gibco, Grand Island, NY, USA) at 27 ℃ and subcultured every 3–4 days. The cells were seeded on a 24-well plate, and after 24 h, the EGFP plasmid was transfected into Sf9 insect cells using Lipofectamine 3000 reagent (Life Technologies, Carlsbad, CA, USA). After 6 h, to activate the pDHsp70 promoter, the cells were treated with heat shock at 42 °C for 30 min. The transfection and expression efficiency of the cells were observed using a fluorescence microscope (Nikon Eclipse Ti, Japan) after 24 h of culture.

Co-immunoprecipitation (Co-IP) plasmid transfection: Sf9 insect cells were seeded on six-well plates 24 h before transfection. The plasmid combinations were co-transfected into the cells using Lipofectamine 300 (for details see Table 1).

**Table 1:** Recombinant plasmid combinations for co-transfection

Recombinant plasmid combinations	Group name	Purpose
pDHsp-70-*LvMmd2*-Flag-His	experimental group 1	CO-IP
pDHsp-70-*LvPAQR3*-ΔC-V5-His		
pDHsp-70-*LvMmd2*-Flag-His	Control group 1-1	
pDHsp-70-V5-His		
pDHsp-70-Flag-His	Control group 1-2	
pDHsp-70-*LvPAQR3*-ΔC-V5-His		
pDHsp-70-*LvPAQR3*-ΔXb-V5-His	Experimental group 2	
pDHsp-70-*LvRaf1*-ΔSTKC-Flag-His		
pDHsp-70-*LvPAQR3*-ΔXb-V5-His	Control group 2-1	
pDHsp-70-Flag-His		
pDHsp-70-V5-His	Control group 2-2	
pDHsp-70-*LvRaf1-*ΔSTKC-Flag-His		
pDHsp-70-*LvMmd2*-Flag-His	Experimental group 3	
pDHsp-70-*LvRho*-V5-His		
pDHsp-70-*LvMmd2*-Flag-His	Control group 3-1	
pDHsp-70-V5-His		
pDHsp-70-Flag-His	Control group 3-2	
pDHsp-70-*LvRho*-V5-His		
pDHsp-70-Flag-His	Control group	
pDHsp-70 -V5-His		
pDhsp-70-*LvMmd2*-EGFP-flag-His	Experimental group	Cell co-location
pDhsp-70-*LvPAQR3*-ΔC-mCherry-V5-His		
pDhsp-70-*LvMmd2*-EGFP-flag-His	Control group 1	
pDhsp-70-mCherry-V5-His		
pDhsp-70-EGFP-flag-His	Control group 2	
pDhsp-70-*LvPAQR3*-ΔC-mCherry-V5-His		
pDhsp-70-EGFP-flag-His	Control group 3	
pDhsp-70-mCherry-V5-His		

Cell co-location plasmid transfection: Sf9 insect cells were seeded on petri dishes 24 h before transfection for cell co-location plasmid transfection. Each respective combination listed in Table 1 was co-transfected. After transfection for 6 h and heat shock at 42 ℃ for 30 min, fluorescence microscope images of *LvMmd2* and *LvPAQR3* distribution patterns were recorded after 24 h of culture.

Golgi staining treatment: Sf9 insect cells were seeded on a petri dish 24 h before transfection. PDhsp-70-*LvMmd2*-EGFP-flag-His and pDhsp-70-EGFP-flag-His plasmids were transfected separately. After transfection for 6 h, heat shock was applied at 42 °C for 30 min, followed by 24 h of culture. The culture medium was then removed, and the cells were washed three times with the pre-cooled Sf-900™ II SFM (Gibco, Carlsbad, CA, USA) culture medium at 4 °C. A diluted Golgi Tracker Red working solution (1:100) was then added to the cells. The cells were incubated in the dark at 4 °C for 30 min, washed three times with the appropriate culture medium at 4 °C, and observed using a fluorescence microscope (Nikon Eclipse Ti, Japan).

### Co-immunoprecipitation (Co-IP) and western blot

After 24 h of pDHsp70 promoter activation, the culture medium was discarded, and the cells were washed three times with PBS. Cell lysis buffer (BeyoTime, Shanghai, China) supplemented with protease inhibitor phenylmethylsulfonyl fluoride (PMSF, 1 mmol/L) was used to lyse the cells and collect proteins. The lysate was then centrifuged at 4 °C at 12,000 rpm for 10 min to remove cell fragments. A quarter of the supernatant (input) was mixed with SDS-PAGE loading buffer and boiled for 10 min to be used as samples to indicate the expression of pDHsp-70-*LvMmd2*-Flag-His, pDHsp-70-*LvPAQR3*-ΔC-V5-His, pDHsp-70-*LvPAQR3*-ΔXb-V5-His, and pDHsp-70-*LvRaf1*-ΔSTKC-Flag-His. The remaining supernatant was subjected to incubation with Anti-FLAG^®^ M2 magnetic beads (Sigma, St. Louis, MO, USA) at 4 °C for 2 h to attract the bait and prey proteins. The magnetic beads were washed twice using PBS and cell lysis buffer. Afterward, they were mixed with 60 μL 2 × SDS-PAGE loading buffer and boiled for 10 min to isolate the proteins from the magnetic beads.

All of the protein samples mentioned above were separated using 15% sodium dodecyl sulfate–polyacrylamide gel electrophoresis (SDS-PAGE) and then transferred onto polyvinylidene difluoride (PVDF) membranes. The protein–protein interactions between pDHsp-70-*LvMmd2*-Flag-His and pDHsp-70-*LvPAQR3*-ΔC-V5-His, pDHsp-70-*LvPAQR3*-ΔXb-V5-His, and pDHsp-70-*LvRaf1*-ΔSTKC-Flag-His were detected using Western blot. The expression of Flag-His-tagged proteins was detected using the Anti-His antibody (rabbit mAb, Cell Signaling Technology, Danvers, MA, USA), and the anti-V5 antibody was used to detect the result of the CO-IP samples and the expression of V5-His-tagged proteins.

### DUAL membrane system yeast two-hybrid assay (MbY2H)

MbY2H was used to identify LvMmd2-interacting proteins. A yeast two-hybrid cDNA was constructed using the Gateway method. Twelve tissues of *L. vannamei* were used as templates: heart, eyestalk, intestine, ventral nerve, hepatopancreas, muscle, brain, stomach, gills, hemocytes, lymphoid organ (Oka), and epidermis. The resulting NubG-X library (pPR3-N library) was constructed by combining it with the NubG fusion cDNA library. The ORF region of *LvMmd2* was cloned into a pBT3-STE vector to construct the bait vector *LvMmd2*-pBT3-STE. Afterward, *LvMmd2*-pBT3-STE and the yeast two-hybrid library of *L. vannamei* were co-transformed into NMY51 and cultured on QDOX-gal 1 mM 3-AT (3-amino-1,2,4-triazole) screening medium. Positive clones were then screened and re-cultured.

The interactions between the obtained positive clones and the *LvMmd2*-pBT3-STE bait protein were supported through one-to-one reactions. The prey plasmid containing the positive clone protein and the bait plasmid *LvMmd2*-pBT3-STE were co-transformed into the NMY51 yeast strain for support. The bait pTSU2-APP, which expresses the type I integral membrane protein APP (amyloid A4 precursor protein), and the prey pNubG-Fe65, which expresses the cytosolic protein Fe65 (amyloid beta A4 precursor protein-binding family B member 1), served as positive controls in the DUAL membrane functional assay. *LvMmd2*-pBT3-STE and pPR3-N, pTSU2-APP and pPR3-N were employed as negative controls. The growth of yeast colonies on SD/-leu/-trp/-his/-ade/X-gal + 1 mM 3-AT medium indicated protein–protein interaction. In addition to the growth reporters HIS3 and ADE2, strain NMY51 also contains the color reporter lacZ, which encodes the bacterial enzyme β-galactosidase. This enzyme catalyzes the conversion of the substrate X-gal into a blue compound, turning yeast cells expressing β-galactosidase blue when incubated with X-gal or a similar substrate. The lacZ reporter gene was used to determine the strength of individual transformant interactions in a library screen.

## Results

### Analysis of genotype and expression of *LvMmd2* in a shrimp family

**Fig. 1:**
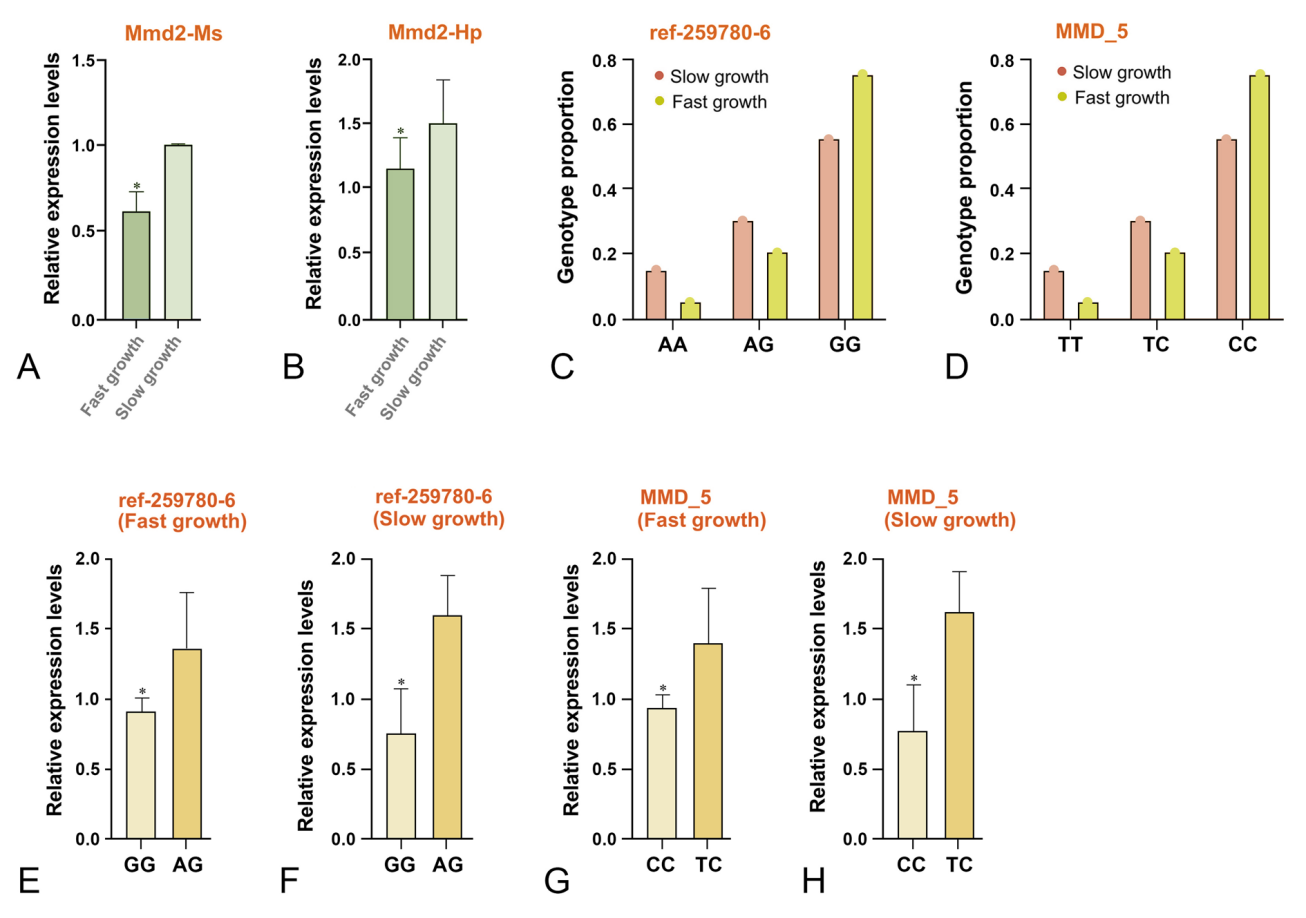
Analysis of genotype and expression of *LvMmd2* in the Family 21253. **A**, **B** Expression analysis of *LvMmd2* in the Family 21253 (*n* = 10). **C** Genotypes of *LvMmd2* at marker ref-259780-6 in Family 21253 (*n* = 40). **D** Genotypes of *LvMmd2* at marker MMD_5 in Family 21253 (*n* = 40). **E** Expression of *LvMmd2* ref-259780-6 marker sites in shrimp with fast growth rate (*n* = 40). **F** Expression of *LvMmd2* ref-259780-6 marker sites in shrimp with slow growth rate (*n* = 40). **G** Expression of *LvMmd2* MMD_5 marker sites in shrimp with fast growth rate (*n* = 40). **H** Expression of *LvMmd2* MMD_5 marker sites in shrimp with slow growth rate (*n* = 40). The results were shown as mean values ± SD. Significant differences of the gene expression levels are shown as *P < 0.05

In this study, slow-growing shrimp (Family 21253) had significantly higher expression of *LvMmd2* than fast-growing shrimp (Fig. 1A, B). The genotype of ref-259780-6 of *LvMmd2* was supported, and the AA and AG types were predominant in the slow-growing shrimp, while the GG type was more common in the fast-growing shrimp (Fig. 1C). Similarly, the support of the MMD_5 marker of *LvMmd2* showed that the TT and CT types were prevalent in the slow-growing shrimp, while the CC type was more common in the fast-growing shrimp (Fig. 1D).

The *LvMmd2* expression level of the dominant genotype shrimp in family 21253 was analyzed. The results indicated that the AG type of LvMmd2 marker ref-259780-6 showed higher expression than the GG type (Fig. 1E, F), while the TC type of MMD_5 marker had higher expression than the CC type (Fig. 1G, H). Moreover, individuals with the GG genotype at ref-259780-6 and the CC genotype at MMD_5 exhibited faster growth rates compared to other genotypes at each marker.

### Expression of growth-related genes after *LvMmd2* knockdown

**Fig. 2:**
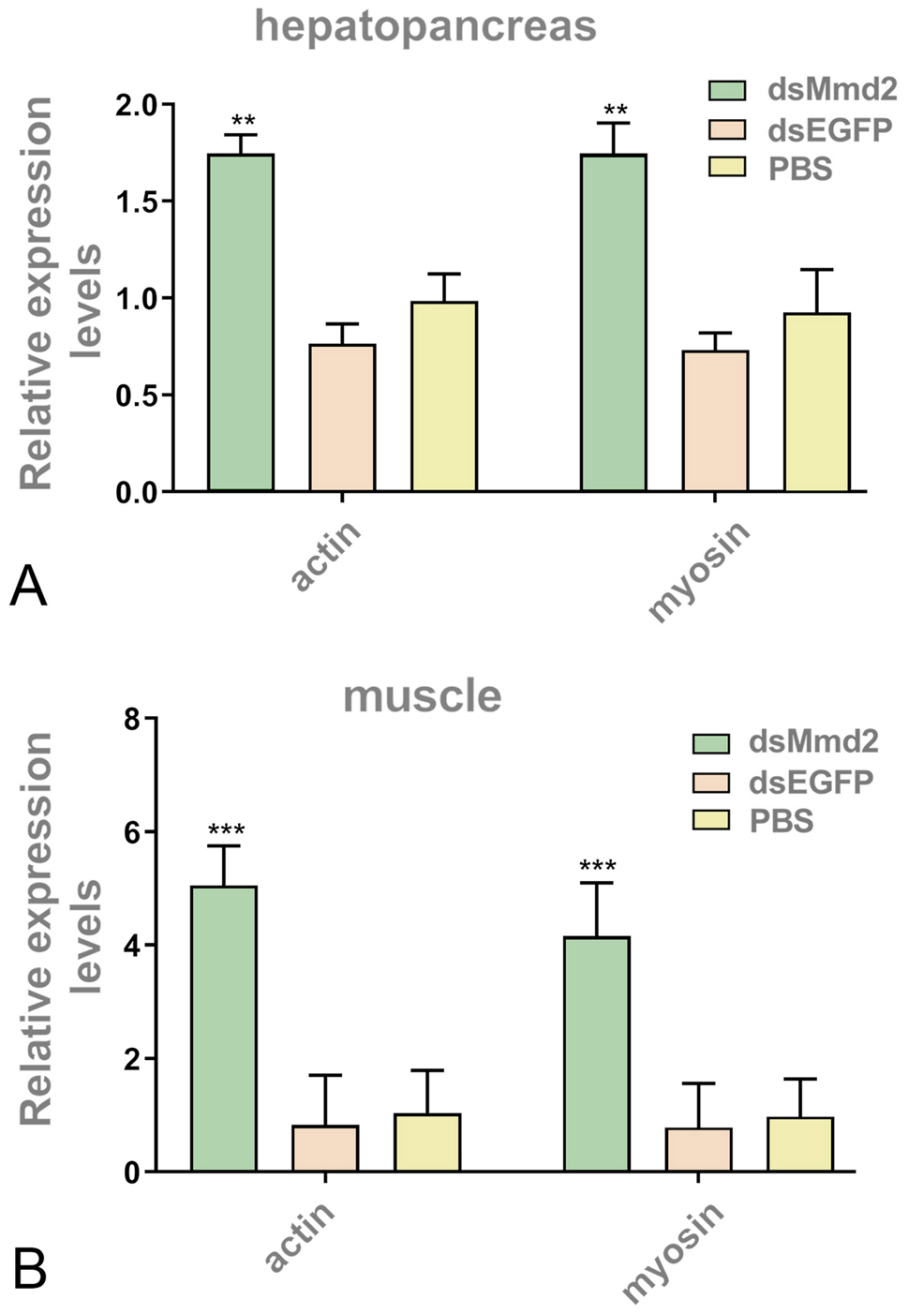
Expression of growth-related genes. **A** Expression of actin and myosin genes in hepatopancreas (*n* = 36). **B** The expression of actin and myosin genes in muscle (*n* = 36). The results were shown as mean values ± SD. Significant differences of the gene expression levels are shown as **P < 0.01 and ***P < 0.001

After *LvMmd2* knockdown, differentially expressed genes (DEGs) in the transcriptome were enriched in growth-related signaling pathways, Ras, Hippo, PI3K/AKT, and insulin signaling pathways (Table 2). In the *LvMmd2* RNAi group, the expression of genes related to the Ras signaling pathway, such as *MRas*, *Rhes*, *Ras-like protein family member 10B*, and *MAPK*, showed significant upregulation. The DEGs were also enriched in the Hippo signaling pathway, showing significant upregulation of the upstream regulatory factors LIX1-like protein and Protein eiger genes. However, the genes in the Hippo signaling pathway (*Mer*, *Hippo*, *STK3*, *Sav*, *LATS1*, *Mats*, *yap1*) showed downregulation, while the downstream effectors (*actin*s) were significantly upregulated (Fig. 2B). In addition, some upregulated DEGs (*AKT*, *IRS*, *Foxo*, and *mTOR*) were also found to be enriched in PI3K/AKT and insulin signaling pathways. Moreover, several immune-related genes, including SVWC domain proteins, c-type lectin domain family 17 (*CLEC17A*), and c-type mannose receptor 2 (*MRC2*), showed significant upregulation following *LvMmd2* knockdown (Table 2). Furthermore, the expression levels of actin and myosin genes, which are responsible for muscle composition, were found to be significantly upregulated in the hepatopancreas and muscle after *LvMmd2* knockdown (Fig. 2A, B).

**Table 2:** Expression of growth-related genes in the transcriptome after *LvMmd2* knockdown

Gene no.	Annotation	Log2(fold change)	Regulation	Related pathways
LOC113800348	Ras-related protein M-Ras	1.08530916696404	Up	Ras signaling pathway
LOC113803395	Ras-like protein family member 10B	0.450762057806748	Up	
LOC113812868	GTP-binding protein Rhes	1.4662980021518	Up	
LOC113804554	Mitogen-activated protein kinase 15	1.3829435959657	Up	
LOC113801201	Mitogen-activated protein kinase 1	0.42690987456159	Up	
LOC113811918 (MZ-ZXJ-Mmd2-AD-7)	Galactose-specific lectin nattectin-like	0.178476	Up	Immune-related gene
LOC113820990 (MZ-ZXJ-Mmd2-AD-16)	Ribonuclease kappa-B-like	− 0.27369	Down	
LOC113807266 (MZ-ZXJ-Mmd2-AD-19)	Single VWC domain protein 3	1.372891304	Up	
LOC113825092	C-type lectin domain family 17	4.032764854	Up	
LOC113804438	C-type mannose receptor 2	2.82362366	Up	
LOC113813212	LIX1-like protein	1.456045	Up	Hippo signaling pathway upstream regulatory genes
LOC113803068	Protein eiger	1.751146	Up	
LOC113801638	Mer	− 0.35699	Down	Hippo signaling pathway-related genes
LOC113816519	hippo	− 0.28407	Down	
LOC113809965	STK3	− 0.094078	Down	
LOC113804778	Sav	− 0.36141	Down	
LOC113801272	LATS1	− 0.05612	Down	
LOC113821688	Mats	− 0.16578	Down	
LOC113804912	yap1	− 0.19537	Down	
LOC113804918	yap1	− 0.072591	Down	
LOC113803355	Actin-57B	4.54572563207977	Up	Hippo signaling pathway downstream effector genes
LOC113803358	Actin-57B	3.54281904464244	Up	
LOC113815117	Actin, muscle	2.15251766783627	Up	
LOC113815142	Actin-2, muscle-specific	3.29156325220765	Up	
LOC113819252	Actin-3, muscle-specific	4.95769196309714	Up	
LOC113812859	RAC serine/threonine-protein kinase(Akt1)	0.0665002261071522	Up	PI3K/AKT signaling pathway and insulin/IGF signaling pathway
LOC113821147	Insulin receptor substrate 1(IRS)	0.405799142101389	Up	
LOC113820016	Forkhead box protein O(FOXO)	0.0575144545810142	Up	
LOC113820097	Serine/threonine-protein kinase mTOR	0.122467578651863	Up

### The role of *LvMmd2* in regulating Ras signaling pathway

**Fig. 3:**
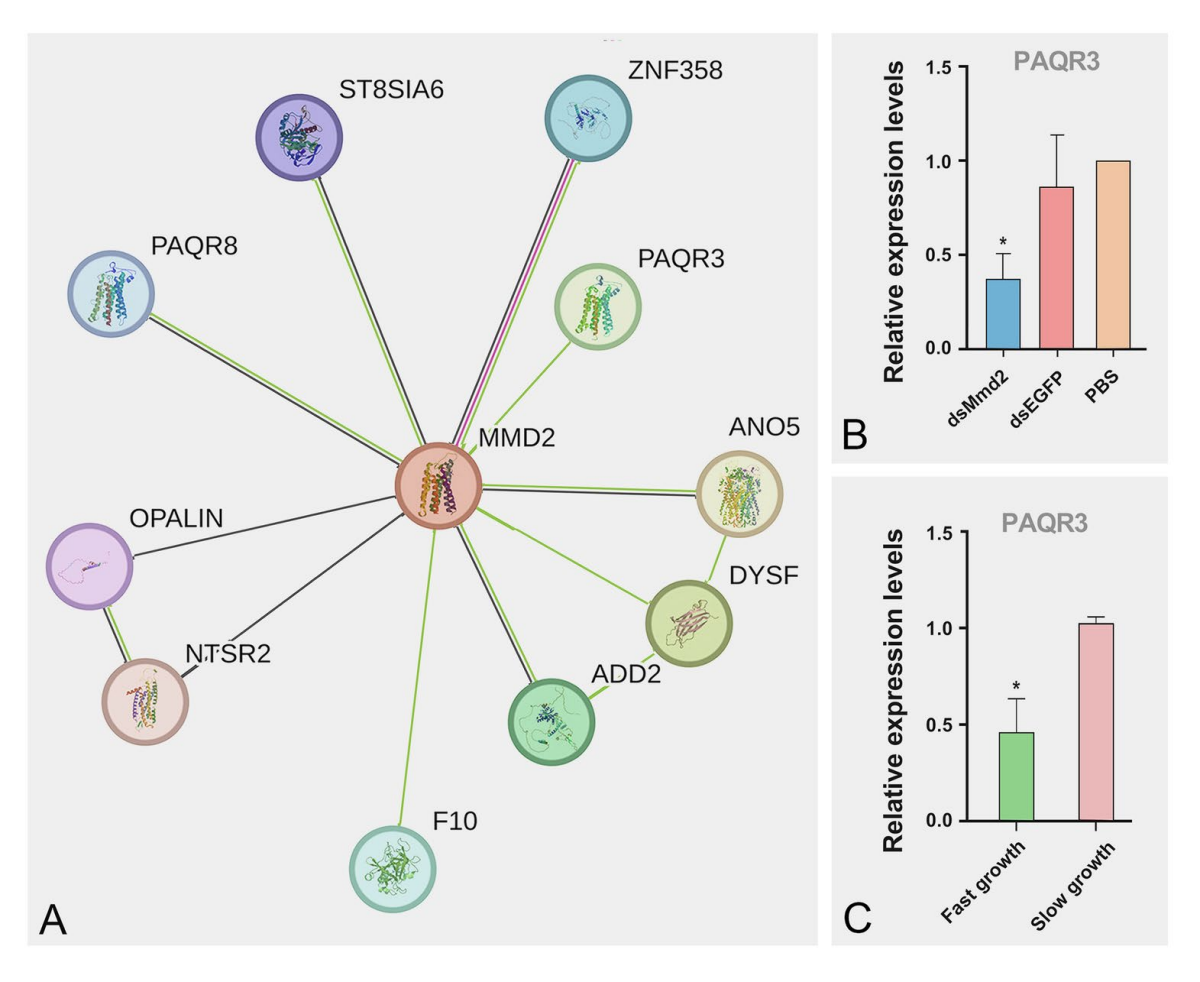
Effect of *LvMmd2* on *LvPAQR3.* **A** Genes predicted to interact with LvMmd2 on String website (https://cn.string-db.org/). **B** Expression of *LvPAQR3* in muscle tissue after dsMmd2 interference (*n* = 36). **C** Expression of *LvPAQR3* in fast- and slow-growing shrimp muscle tissue of Family 21253 (*n* = 10). The results were shown as mean values ± SD. Significant differences of the gene expression levels are shown as *P < 0.05

**Fig. 4:**
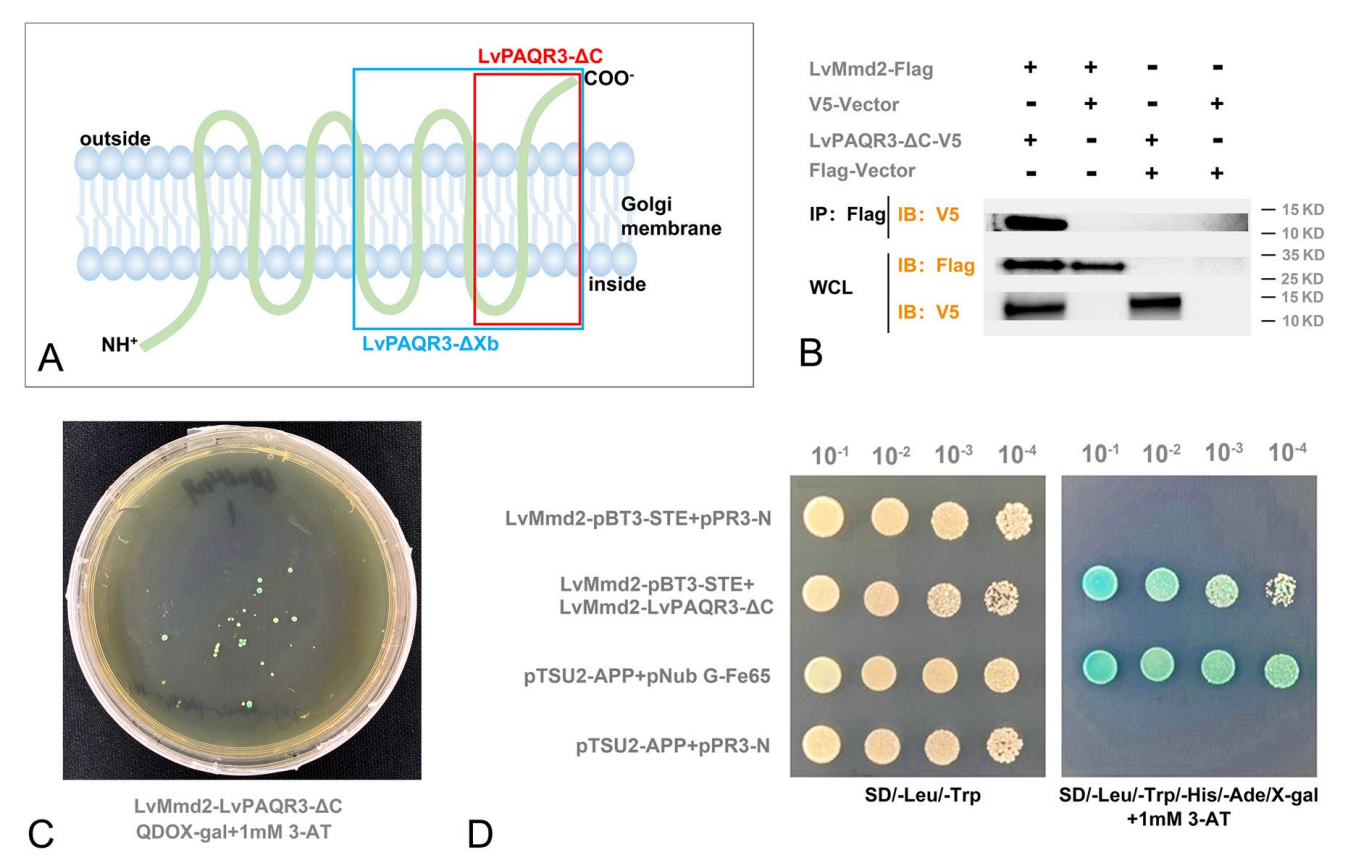
Interaction of LvMmd2 with its homolog LvPAQR3. **A** Protein structure model of *LvPAQR3*. **B** Co-immunoprecipitation assay of LvMmd2 and LvPAQR3-ΔC. **C** Yeast two-hybrid assay of LvMmd2 and LvPAQR3-ΔC. **D** One-to-one validation of yeast two-hybrid assay of LvMmd2 and LvPAQR3-ΔC

STRING predicted that there was an interaction between LvMmd2 (PAQR10) and its homologous protein LvPAQR3 (Fig. 3A). The results of expression analysis showed significant downregulation of *LvPAQR3* expression after *LvMmd2* knockdown (Fig. 3B). In addition, it was observed that shrimp with a slow growth rate had higher expression of *LvPAQR3* compared to those with a fast growth rate in Family 21253 (Fig. 3C). The co-immunoprecipitation results showed an interaction between LvMmd2 and LvPAQR3-ΔC (Fig. 4A, B). Furthermore, the interaction between LvMmd2 and LvPAQR3-ΔC was also supported through MbY2H (Fig. 4C). Yeast cells expressing β-galactosidase turned blue when incubated with X-gal or a similar substrate (Fig. 4D).

**Fig. 5:**
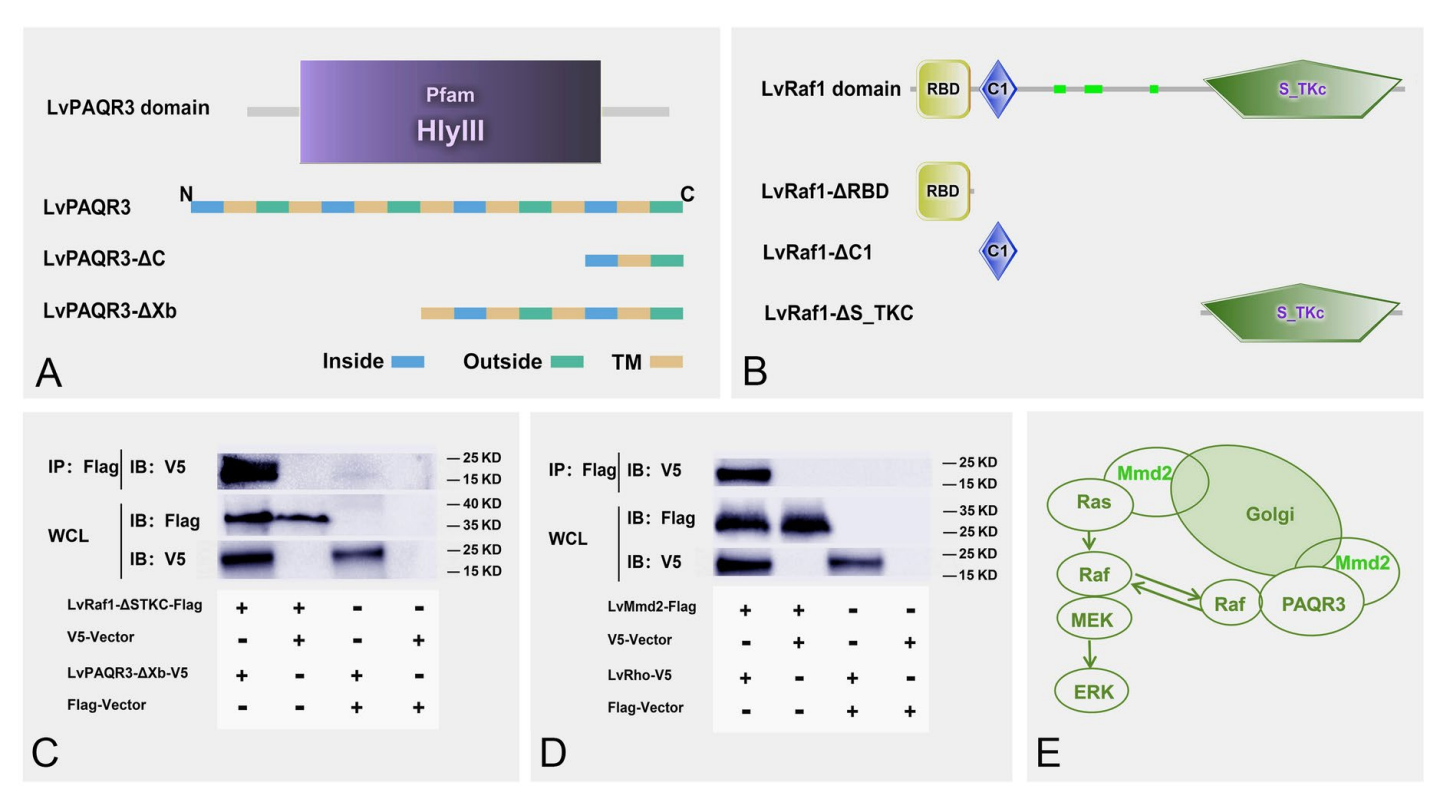
*LvMmd2* affects Ras signaling pathway. **A** Protein structure model of LvPAQR3. **B** Protein structure model of LvRaf1. **C** Co-immunoprecipitation result of interaction between LvRaf1-ΔSTKC and LvPAQR3-ΔXb. **D** Co-immunoprecipitation result of interaction between LvMmd2 and LvRho. **E** *LvMmd2* directly or indirectly regulates Ras signaling pathway

The co-immunoprecipitation result showed that LvPAQR3-ΔXb interacted with LvRaf1-ΔSTKC (Fig. 5C). The *LvRho* gene, which was significantly downregulated in the *LvMmd2* RNAi transcriptome, was cloned and expressed in Sf9 insect cells. The co-immunoprecipitation result showed an interaction between LvRho and LvMmd2 (Fig. 5D), indicating that LvMmd2 can directly affect the Ras signaling pathway by interacting with LvRho (Fig. 5E).

### Subcellular co-location of LvMmd2 and LvPAQR3

**Fig. 6:**
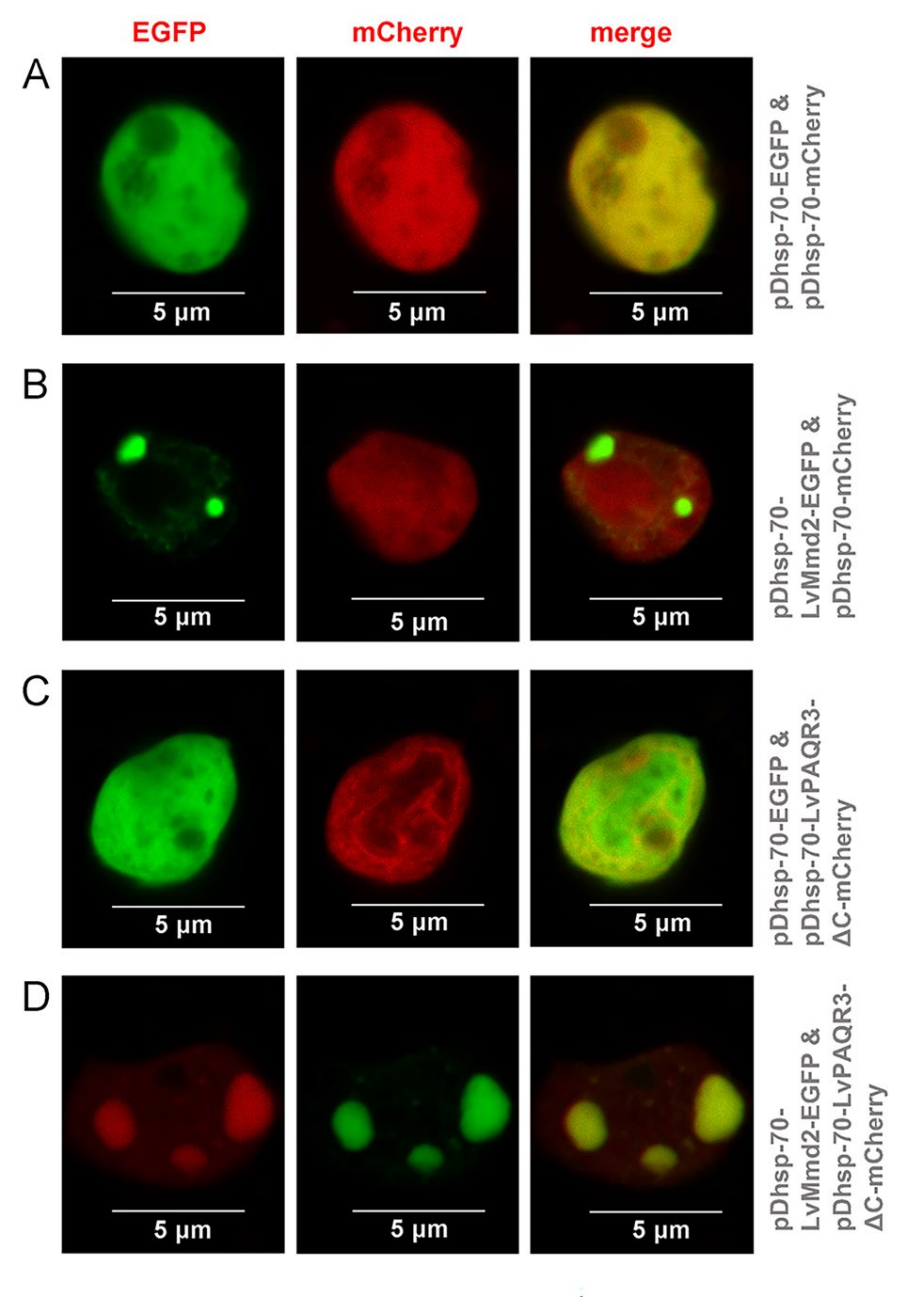
Subcellular co-location of LvMmd2 and LvPAQR3. **A** Subcellular location of EGFP and mCherry. **B** Subcellular location of LvMmd2 and mCherry. **C** Subcellular location of EGFP and LvPAQR3. **D** Subcellular location of LvMmd2 and LvPAQR3

The results of subcellular co-location further investigated the subcellular localization of LvMmd2 and LvPAQR3. In the negative control group, the green and red fluorescence signals expressed by pDhsp-70-EGFP-flag-His and pDhsp-70-mCherry-V5-His, respectively, were evenly distributed in the cytoplasm of sf9 insect cells (Fig. 6A) However, LvMmd2-EGFP protein fluorescence signals were found to aggregate into bright spots in sf9 insect cells by co-transfecting with pDhsp-70-LvMmd2-EGFP-flag-His and pDhsp-70-mCherry-V5-His (Fig. 6B), while the mCherry fluorescence signals were still evenly distributed in the cytoplasm. When pDhsp-70-EGFP-flag-His and pDhsp-70-LvPAQR3-ΔC-mCherry-V5-His were co-transfected into sf9 insect cells, the fluorescence signals were evenly distributed in the cytoplasm, similar to the negative control (Fig. 6C). However, when pDhsp-70-LvMmd2-EGFP-flag-His and pDhsp-70-LvPAQR3-ΔC-mCherry-V5-His were co-transfected, the green and red fluorescence signals aggregated into bright spots in sf9 insect cells (Fig. 6D).

LvMmd2-EGFP was observed to be distributed in dots in the sf9 insect cells, which exhibited a pattern similar to the Golgi labeled with Golgi Tracker Red (Supplementary Fig. S3).

### Other genes interacting with *LvMmd2*

*LvMmd2*-pBT3-STE and the yeast library from *L. vannamei* were co-transformed into the yeast strain NMY51, and a total of 21 positive candidate clones were obtained (Supplementary Table S6). The annotations of these clones were summarized into nine candidate genes (Table 3). These genes were further indicated to interact with LvMmd2 through one-to-one rotation support with *LvMmd2*-pBT3-STE (Supplementary Fig. S1). The genes include *myotrophin-like*, *cuticular protein 47Eg-like*, *galactose-specific lectin nattectin-like*, *ribonuclease kappa-B-like*, *single VWC domain protein*, *translocon-associated protein subunit gamma-like*, *signal peptidase complex subunit 2 isoform X2*, *rhodopsin* and *NADH-ubiquinone oxidoreductase chain 1-like*.

**Table 3:** The nine positive clones interacting with LvMmd2 screened by yeast two-hybrid assay

Gene number	Gene name	*E*_value	Genbank accession no.	Pairwise QDOX-gal + 1 mM 3-AT
Mmd2-AD-7	Galactose-specific lectin nattectin-like	2.42854E−63	XP_027219573.1	Blue
Mmd2-AD-8	Myotrophin-like	Inf	XM_027355709.1	Blue
Mmd2-AD-13	Cuticular protein 47Eg-like	1.69273E−34	XP_027213691.1	Blue
Mmd2-AD-16	Ribonuclease kappa-B-like	4.73362E−58	XP_027229213.1	Blue
Mmd2-AD-18	Translocon-associated protein subunit gamma-like	4.6811E-111	XP_027232600.1	Blue
Mmd2-AD-19	Single VWC domain protein	6.01705E−67	XP_027214279.1	Blue
Mmd2-AD-20	Signal peptidase complex subunit 2 isoform X2	2.3967E−134	XP_027214317.1	Blue
Mmd2-AD-22	Rhodopsin	0	ROT66570.1	Blue
Mmd2-AD-26	NADH-ubiquinone oxidoreductase chain 1-like	3.0659E−172	XP_027228409.1	Blue

## Discussion

### *LvMmd2* is a growth-inhibiting gene

*LvMmd2*, identified as a growth-related gene (Wang et al. 2020), is a conserved membrane protein gene whose knockdown significantly increases shrimp growth, indicating its inhibitory role (Si et al. 2023). Here, we explored the molecular mechanism by which *LvMmd2* regulates shrimp growth by validating its expression and genotyping within a family with varying growth rates. We observed higher levels of *LvMmd2* expression were observed in the slow-growing group, with the AG/TC genotype predominating in this group and GG/CC being more common in the fast-growing group. This supports the prediction of *LvMmd2* is a growth-inhibiting gene (Si et al. 2023). Since marker ref-259780-6 is located in the intron region of *LvMmd2* and MMD_5 is located in the fourth exon of *LvMmd2*, further research is needed to elucidate the relationship between the location of causative loci and *LvMmd2* expression.

### *LvMmd2* affects Ras signaling pathway

In our previous study, we identified the Ras signaling pathway as a critical pathway affected by *LvMmd2* knockdown (Si et al. 2023). To explore the impact of *LvMmd2* on Ras signaling pathway in shrimp, we conducted protein interaction assays and found a potential interaction between Mmd2 and its homolog PAQR3. Furthermore, *LvPAQR3* expression decreased after *LvMmd2* knockdown (Fig. 3B). Moreover, *LvPAQR3* expression was significantly higher in slow-growing shrimp compared to their fast-growing counterparts. CO-IP and MbY2H results indicated the interaction between LvMmd2 and LvPAQR3 at both molecular and cellular levels. Fluorescence co-localization studies showed LvMmd2 increased the retention of LvPAQR3-ΔC in the Golgi apparatus, suggesting an interaction that promotes its aggregation. Previous research has demonstrated that PAQR3 regulates the Ras signaling pathway by trapping Raf1 (Fan et al. 2008; Feng et al. 2007). Our CO-IP results further revealed that LvPAQR3-ΔXb interacts with LvRaf1-ΔSTKC in sf9 insect cells. In conclusion, LvMmd2 interacts with LvPAQR3, and LvPAQR3 interacts with LvRaf1. Therefore, LvMmd2 may affect the capture of Raf1 by interacting with LvPAQR3, thus indirectly regulating the Ras signaling pathway in shrimp.

Besides its indirect effect through PAQR3, *LvMmd2* may directly influence the Ras signaling pathway in shrimp. Jin et al. (2012a) have reported the interaction between PAQR10 and Ras in the human cell Golgi apparatus, leading to the activation of the ERK signaling pathway. This discovery has identified PAQR10 as a new class of Ras modulators. In mammals, Mmd2 directly interacts with the three Ras family members HRas, NRas, and KRas4A in regulating the Ras signaling pathway (Jin et al. 2012b). Ras, as a major upstream regulator, plays a crucial role in maintaining organismal homeostasis (Khan et al. 2019). Interestingly, our study found an interaction between LvMmd2 and LvRho (a member of the Ras superfamily). LvRho, which is significantly downregulated after *LvMmd2* knockdown, is known to regulate the actin cytoskeleton (Khosravi-Far and Der 1994). These results suggest that *LvMmd2* may also regulate shrimp growth by directly affecting the Ras signaling pathway.

### *LvMmd2* affects shrimp growth through PAQR3 and Raf1

*PAQR3* plays crucial roles in growth-related signaling pathways and is a key regulator of inflammation and metabolism, negatively regulating the PI3K/AKT signaling pathway. PI3K/AKT, central to the insulin signaling pathway, regulates glucose and lipid metabolism (Xiao et al. 2022). In diabetic patients, *PAQR3* is upregulated, which shunts p110a to the Golgi apparatus, preventing p110a from forming a PI3K complex with p85a and thus negatively regulating the insulin signaling pathway (Qiao et al. 2021). In this study, when *LvMmd2* was knocked down, the expression of *LvPAQR3* also decreased (Fig. 3B), and the genes related to PI3K/AKT and insulin signaling pathway were upregulated, such as *Akt*, *IRS*, *Foxo*, and *mTOR* (Table 2). These findings may explain why *LvMmd2* affects the growth of *L. vannamei*.

As a homolog of Mmd2 (PAQR10), PAQR3 has been reported to regulate the Ras signaling pathway by recruiting Raf1 (Feng et al. 2007). When *LvPAQR3* expression decreases, more *LvRaf1* may be released to participate in other signaling pathways. Raf1 may also play an important role in the growth of organisms. *Raf1* is a negative regulator of apoptosis (Nolan et al. 2021). *Raf1* can inhibit apoptosis in two different activation states: by binding *MST2* when inactivated and by binding *ASK1* when activated (Alavi et al. 2007; Dhillon et al. 2002). *Raf1* was found to inhibit *ASK1* pro-apoptotic signaling through overexpression (Chen et al. 2001). Moreover, Raf1 can bind to and inhibit the activation of MST2 kinase, thereby suppressing *MST2*-mediated apoptosis (O'Neill et al. 2005). *MST2* kinase serves as an upstream regulator of Hippo signaling pathway to inhibit cell growth (Aihara et al. 2022; García-Gutiérrez et al. 2022). In the *LvMmd2* RNAi transcriptome, the upregulation of upstream inhibitor genes of the Hippo signaling pathway, *Lft* (*Lix1*) and *Egr*, suggests pathway inhibition. The downregulation of *Mer*, *Hippo (hpo*, *STK3*), *Salvador* (*sav*), *Warts* (*LATS1*), *Mats*, and *Yki* (*Yap1*) further indicates suppressed Hippo signaling pathway after *LvMmd2* knockdown (Table 2). In addition, Raf1 was reported to promote cell survival by inactivating BAD through direct phosphorylation (Wang et al. 1996). It acts as an adaptor protein, facilitating BAD binding to protein kinase theta (PKCθ), which phosphorylates and subsequently inactivates BAD (Hindley and Kolch 2007). Therefore, Raf1 can inhibit cell apoptosis through three mechanisms.

In addition, Raf1 is involved in various signaling pathways related to cell growth. It facilitates cell proliferation and migration, as well as protects cells from DNA damage. Raf1 interacts with ROK-α, inhibiting its kinase activity, which subsequently activates STAT3 and MYC, leading to cell dedifferentiation (Nolan et al. 2021). Conversely, the binding of RHO to ROK-α alleviates Raf1’s inhibitory effect, thereby promoting cell migration and differentiation through EZRIN (Ehrenreiter et al. 2005, 2009). Unlike its inhibitory interactions with MST2, ASK1, or ROK-α, Raf1 enhances PLK1 activation, thus promoting cell division (Nolan et al. 2021). Besides, Raf1 binds to the Ser/Thr kinase CHK2, promoting DNA repair and safeguarding cells from DNA damage (Zannini et al. 2014).

**Fig. 7:**
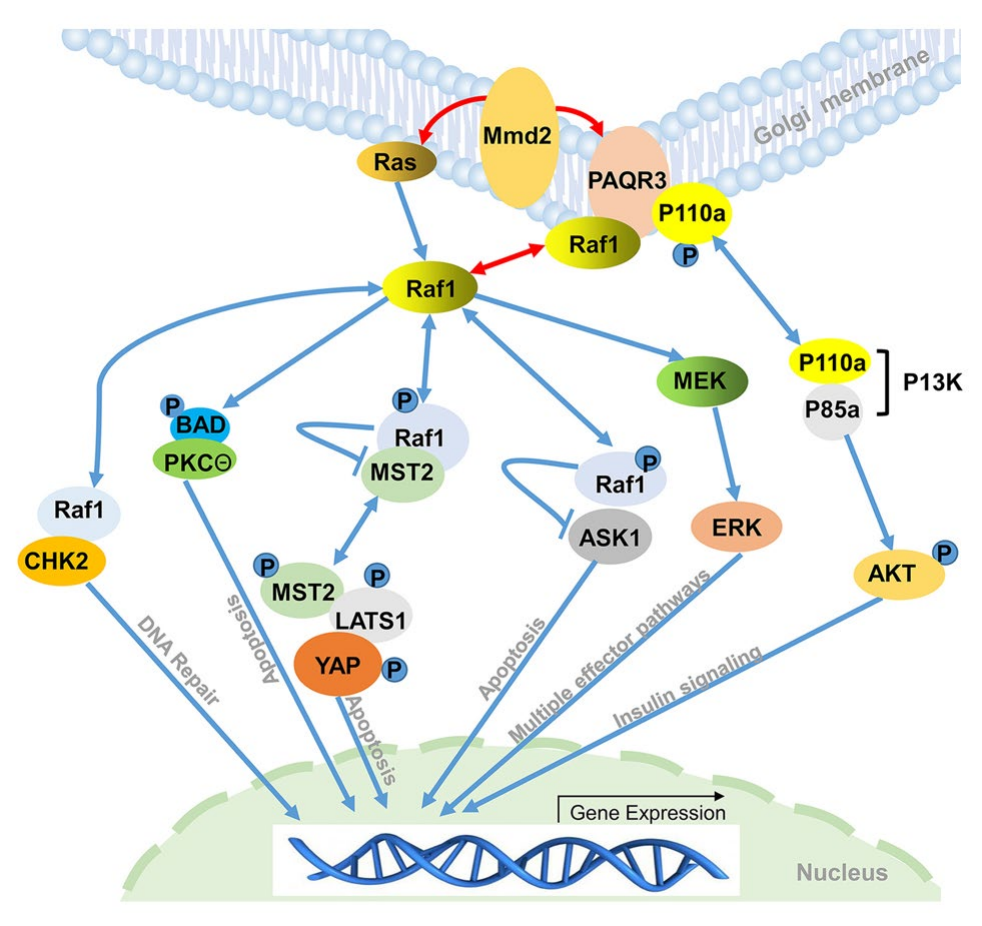
Deduced signaling pathways regulated by LvMmd2. The blue lines indicate that the relationship was demonstrated by other researchers, and the red lines indicate that the relationship was demonstrated in this study

In conclusion, our results suggest that *LvMmd2* not only controls shrimp muscle growth through PAQR3 and Raf1 but also regulates various cellular processes, such as cell proliferation, migration and differentiation, as well as protects cells from DNA damage (Fig. 7).

### *LvMmd2* affects the shrimp growth through other aspects

Our previous transcriptome analyses suggested that *LvMmd2* may affect growth, immunity, and metabolism in shrimp (Si et al. 2023). In the present study, the MbY2H results indicated that LvMmd2 interacts with multiple proteins related to shrimp growth and immunity. Among them, myotrophin and cuticular protein are associated with muscle formation, growth, and molting in shrimp. Galactose-specific lectin nattectin-like, ribonuclease kappa-B-like, and single VWC domain protein are related to the immune response of shrimp. Translocon-associated protein subunit gamma-like and signal peptidase complex subunit 2 play roles in protein transport, synthesis, and modification. In addition, LvMmd2 also interacts with rhodopsin and NADH-ubiquitin oxidoreductase chain 1-like.

*LvMmd2* not only enhances shrimp growth and suppresses cell apoptosis by interacting with Raf1, Ras, and Hippo signaling pathways, but also has connections with other genes related to shrimp growth. In Simmental beef cattle, *myotrophin* has been identified as a candidate gene for muscle development and net meat weight (NMW). It promotes skeletal muscle cell differentiation and skeletal muscle hypertrophy (Bordbar et al. 2020). Porcine *S-myotrophin* leads to the hypertrophy of skeletal muscle cells through the accumulation of muscle structural proteins (Hayashi et al. 1998, 2001). In addition, *S-myotrophin* can also increase the production of fast myosin in mice (Shiraishi et al. 2006). In *L. vannamei*, LvMmd2 interacts with myotrophin, potentially increasing the accumulation of muscle structural proteins and promoting the growth of muscle fibers in shrimp. It was observed in the *LvMmd2* RNAi transcriptome that multiple actin and myosin genes were significantly upregulated (Fig. 2).

The cuticle is primarily composed of chitin and cuticular proteins (CPs) (Pan et al. 2018). The CPs influence cuticle structure during insect growth, reproduction, and environmental adaptation processes (Xie et al. 2022). In the *LvMmd2* RNAi transcriptome*,* the significantly upregulated gene enrichment reveals biological functions in chitin metabolism and morphogenesis (Si et al. 2023). The results of MbY2H demonstrated the interaction between LvMmd2 and CPs, suggesting that *LvMmd2* potentially affects cuticle formation and chitin metabolism in shrimp.

LvMmd2 may also affect shrimp growth by regulating the immune system. C-type lectin-nattectin-like protein (CaNTC) binds to pathogen-associated molecular patterns (PAMPs) (Lopes-Ferreira et al. 2021) and participates in antibacterial and antiviral immunity (Wang et al. 2017). EsSVWC helps to resist bacterial infection and improve survival (Qin et al. 2022). Ribonuclease kappa-B-like (*Rnasek-b*) is specifically expressed in hepatopancreas, gills, and hemocytes, which are all immune-related organs in *L. vannamei* (Supplementary Fig. S2). In our MbY2H results, the galactose-specific lectin nattectin-like, the ribonuclease kappa-B-like, and a single VWC domain protein interacted with LvMmd2. The expression of these three genes was significantly upregulated after *LvMmd2* knockdown. In addition, many other immune-related genes, such as C-type lectin family member 17 and C-type mannose receptor 2, were also significantly upregulated in the *LvMmd2* RNAi transcriptome (Table 2). These results indicate that LvMmd2 is involved in shrimp immunity. Therefore, LvMmd2 may also impact shrimp growth by regulating the immune system.

LvMmd2 also interacts with proteins involved in protein transport, synthesis, and modification, such as translocon-associated protein subunit gamma-like (*TRAPγl*). The translocon-associated protein (*TRAP*) complex facilitates the insertion or translocation of newly synthesized proteins in eukaryotic cells (Li et al. 2005). In addition, LvMmd2 may also be present in the Golgi apparatus, which serves as the primary location for protein processing and modification. Therefore, LvMmd2 may interact with the translocon-associated protein and the signal peptidase complex subunit 2, contributing to protein processing and modification in the Golgi apparatus.

In this study, we found that *LvMmd2* regulates the growth of *L. vannamei* through multiple mechanisms, including the Ras, Hippo, and insulin signaling pathways, which are crucial in crustacean biological processes. Understanding the role of *Mmd2* in these pathways is conducive to gradually elucidating the regulation of the Ras, Hippo, and insulin signaling pathways on shrimp growth. Through further research, we hope to uncover more about the mechanisms of the Mmd2 gene in the growth and development of *L. vannamei* and apply this knowledge to improve the growth efficiency of shrimp.

## Conclusion

This study provides a preliminary analysis of the molecular mechanisms through which *LvMmd2* regulates the growth of *L. vannamei*. LvMmd2 is found to be located in the Golgi apparatus, where it interacts with LvPAQR3 and LvRho. Furthermore, LvPAQR3 interacts with LvRaf1. LvMmd2 is involved in regulating the Ras, PI3K/AKT, Hippo, and insulin signaling pathways, ultimately affecting cell proliferation and differentiation. Moreover, the interaction between LvMmd2 and other proteins related to immunity, growth, and protein modification suggests that it regulates growth through multiple mechanisms. These findings lay a foundation for further studies on the role of the *Mmd2* gene in the growth of shrimp and crustaceans in general.

## Supplementary information

Below is the link to the electronic supplementary material.

Supplementary file1 (DOCX 10731 KB)

## Data Availability

The data presented in the study are deposited in the NCBI Sequence Read Archive (https://www.ncbi.nlm.nih.gov/sra), accession numbers: PRJNA918508, SRR22981843-SRR22981848.
