# Supplementary material for: Molecular mechanisms of Mmd2 gene in regulating growth of the Pacific white shrimp Litopenaeus vannamei
Source: Mar Life Sci Technol. 2025 Feb 15;7(1):50–65. doi: 10.1007/s42995-024-00273-7 (PMC11871217; doi:10.1007/s42995-024-00273-7)
Supplement: Supplementary file 1 — Supplementary file1 (DOCX 10731 KB) [file 42995_2024_273_MOESM1_ESM.docx]

***Supplementary Material***

**Molecular mechanisms of Mmd2 gene in regulating growth of shrimp**

Shuqing Si^1,2,3^, Xiaojun Zhang^1,2,3*^, Yang Yu^1,2,3^, Xiaoyun Zhong^1,2,3^, Xiaoxi Zhang^1,2,3^, Jianbo Yuan^1,2,3^, Ka Hou Chu^5,6^, Fuhua Li^1,2,3,4^

^1^ CAS and Shandong Province Key Laboratory of Experimental Marine Biology, Center for Ocean Mega-Science, Institute of Oceanology, Chinese Academy of Sciences, Qingdao 266071, China

^2^ Laboratory for Marine Biology and Biotechnology, Qingdao Marine Science and Technology Center, Qingdao 266237, China

^3^ College of Earth Science, University of Chinese Academy of Sciences, Beijing 100049, China

^4^ Key Laboratory of Breeding Biotechnology and Sustainable Aquaculture, Chinese Academy of Sciences, Wuhan 430072, China

^5^ Southern Marine Science and Engineering Guangdong Laboratory (Guangzhou), Guangzhou 510301, China

^6^ School of Life Sciences, The Chinese University of Hong Kong, Shatin, NT, Hong Kong, China

* Corresponding author: e-mail address: xjzhang@qdio.ac.cn (Xj Zhang)

**1 Supplementary Tables**

Table S1 The primers were designed for Real-time Quantitative PCR experiments.

| Primer ID | Primer sequence (5'-3') | | Fragment length (bp) | Ta (℃) |
| --- | --- | --- | --- | --- |
| RT-*LvPAQR3* | Forward | CTTAGTATGTGTCCTGTTTAGCG | 187 | 58 |
|  | Reverse | CATACATCATCAATATACGGGGC |  |  |
| RT-*actin* | Forward | CCTTGACTTCGAGAGTGAGATGA | 165 | 58 |
|  | Reverse | GTCTCATGAACACCAGCAGATTC |  |  |
| RT-*myosin* | Forward | CAACCCTATTCTTGAAGCCTTCG | 194 | 57 |
|  | Reverse | AAGATGTGGAATGTGCGTTCATC |  |  |
| RT-*LvMmd2* | Forward | GCAGCCTTTATCCATTACTATGC | 202 | 57 |
|  | Reverse | GCAGCCTTTATCCATTACTATGC |  |  |

Table S2 The primers are designed for all gene cloning.

| Primer ID | Primer sequence (5'-3') | | Fragment length (bp) | Ta (℃) |
| --- | --- | --- | --- | --- |
| *LvPAQR3* | Forward | GATCTTTTGACGCTGTGATAAGAG | 1090 | 59 |
|  | Reverse | CCAGTGCAGACTTATTATTTTCCC |  |  |
| *LvRaf1* | Forward | CGCACTAATTTTGGCGGATGATA | 2660 | 60 |
|  | Reverse | GTCTAGTAGAGTCTCCTTTGGGG |  |  |
| *LvRho* | Forward | ATGAAGTGGAGAAGCCGTGAATC | 369 | 56 |
|  | Reverse | AATAAATGGTCCCAAAGTGACTACCTC |  |  |
| pDhsp-70-mCherry | Forward | GTACCGAGCTCGGATCCATGGTGAGCAAGGGCGAGGAGGAT | 718 | 57 |
|  | Reverse | GGCCCTCTAGACTCGAGCTTGTACAGCTCGTCCATGCCG |  |  |

Table S3 The primers are designed for plasmid construction.

| Primer ID | Primer sequence (5'-3') | | Fragment length (bp) | Ta (℃) |
| --- | --- | --- | --- | --- |
| pDHsp-70-*LvMmd2* | Forward | GTTACCGAGGAAGCTTATGGTCATGGCTTGCCG | 651 | 57 |
|  | Reverse | CTGGACTAGTGGATCCCAAGCTAGAGCACTCAGGCA |  |  |
| pDHsp-70-*LvPAQR3*-ΔC | Forward | GTTACCGAGGAAGCTTTGGAAAATACCAGAGAGATATTTTCC | 171 | 57 |
|  | Reverse | CTGGACTAGTGGATCCTTCCTTTGCAGAAATGCCACTA |  |  |
| pDHsp-70-*LvPAQR3*-xb-Δxb | Forward | GTTACCGAGGAAGCTTGGATTTTGGTGCCCTGAG | 592 | 59 |
|  | Reverse | TTGGTGCCCTGAGTTATTACATGGATCCACTAGTCCAG |  |  |
| pDHsp-70-*LvRaf*-ΔSTKC | Forward | GTTACCGAGGAAGCTTATGATCCTGATTGGTCATCGC | 794 | 58 |
|  | Reverse | CTGGACTAGTGGATCCTAAGTTGGCTAGGATCTGTTGG |  |  |
| pDHsp-70-*LvRho* | Forward | GTTACCGAGGAAGCTTATGAAGTGGAGAAGCCGTGAATC | 373 | 56 |
|  | Reverse | GAGGTAGTCACTTTGGGACCATTTATTGGATCCACTAGTCCAG |  |  |
| pDHsp-70-*LvMmd2*-EGFP | Forward | ACAAGTCGTTACCGAGGAAGCTTATGGTCATGGCTTGCCGCTCTG | 1390 | 59 |
|  | Reverse | TCGCCCTTGCTCACCATGGATCCCAAGCTAGAGCACTCAGGCAAAG |  |  |
| pDHsp-70-*LvPAQR3*-ΔC-mCherry | Forward | ACAAGTCGTTACCGAGGAAGCTTATGTGGAAAATACCAGAGAGAT | 898 | 60 |
|  | Reverse | TCGCCCTTGCTCACCATGGATCCTTCCTTTGCAGAAATGCCACTAC |  |  |

Table S4 The recombinant plasmids.

| recombinant plasmid | Fragment length (bp) | Carrier | restriction enzyme | Primer ID |
| --- | --- | --- | --- | --- |
| pDHsp-70-*LvMmd2*-Flag-His | 786 | pDHsp-70-Flag-His vector | HindⅢ | pDHsp-70-*LvMmd2* |
|  |  |  | BamHI |  |
| pDHsp-70-*LvPAQR3*-Δxb-V5-His | 612 | pDHsp-70-V5-His vector | HindⅢ | pDHsp-70-*LvPAQR3*-Δxb |
|  |  |  | BamHI |  |
| pDHsp-70-*LvPAQR3*-ΔC-V5-His | 327 | pDHsp-70-V5-His vector | HindⅢ | pDHsp-70-*LvPAQR3*-ΔC |
|  |  |  | BamHI |  |
| pDHsp-70-*LvRaf1*-ΔSTKC-Flag-His | 591 | pDHsp-70-Flag-His vector | HindⅢ | pDHsp-70-*LvRaf1*-ΔSTKC |
|  |  |  | BamHI |  |
| pDHsp-70-*LvRho*-V5-His | 510 | pDHsp-70-V5-His vector | HindⅢ | pDHsp-70-*LvRho* |
|  |  |  | BamHI |  |
| pDhsp-70-EGFP-flag-His | 831 | pDHsp-70-Flag-His vector | BamHI | pDhsp-70-EGFP |
|  |  |  | Xho I |  |
| pDhsp-70-mCherry-V5-His | 841 | pDHsp-70-V5-His vector | BamHI | pDhsp-70-mCherry |
|  |  |  | Xho I |  |
| pDhsp-70-*LvMmd2*-EGFP-flag-His | 1488 | pDHsp-70-Flag-His vector | HindⅢ | pDhsp-70-*LvMmd2* |
|  |  |  | BamHI |  |
| pDhsp-70-*LvPAQR3*-ΔC-mCherry-V5-His | 1018 | pDHsp-70-V5-His vector | HindⅢ | pDhsp-70-*LvPAQR3*-ΔC |
|  |  |  | BamHI |  |

Table S5 The primers designed for the identification of SNP in the coding region of *LvMmd2.*

| Primer ID | Primer sequence (5'-3') | | Fragment length (bp) | Ta (℃) |
| --- | --- | --- | --- | --- |
| Primer-Mmd2-M1 | Forward | AATCCACAGTTCACGCAACTAGA | 106 | 58 |
|  | Reverse | CAAAACTGCCTTACTGCTCCATA |  |  |
| Primer-Mmd2-M4 | Forward | ACAATCGCTTCAACCAACCTG | 270 | 58 |
|  | Reverse | TCCTGGGCGTGATCTTCTTT |  |  |

Table S6 The 21 positive clones of yeast two-hybrid.

| gene number | gene name | E_value | genebank |
| --- | --- | --- | --- |
| Mmd2-AD-13 | cuticular protein 47Eg-like | 1.69273E-34 | XP_027213691.1 |
| Mmd2-AD-16 | ribonuclease kappa-B-like | 4.73362E-58 | XP_027229213.1 |
| Mmd2-AD-18 | translocon-associated protein subunit gamma-like | 4.6811E-111 | XP_027232600.1 |
| Mmd2-AD-27 |  | 3.4519E-111 |  |
| Mmd2-AD-19 | single VWC domain protein 3 | 6.01705E-67 | XP_027214279.1 |
| Mmd2-AD-20 | signal peptidase complex subunit 2 isoform X2 | 2.3967E-134 | XP_027214317.1 |
| Mmd2-AD-4 |  | 2.9115E-134 |  |
| Mmd2-AD-5 |  | 3.1366E-134 |  |
| Mmd2-AD-24 | signal peptidase complex subunit 2 isoform X1 | 2.0615E-136 | XP_027214316.1 |
| Mmd2-AD-22 | rhodopsin | 0 | ROT66570.1 |
| Mmd2-AD-26 | NADH dehydrogenase subunit 1 | 3.0659E-172 | YP_001315044.1 |
| Mmd2-AD-7 | galactose-specific lectin nattectin-like | 2.42854E-63 | XP_027219573.1 |
| Mmd2-AD-8 | myotrophin-like |  | XM_027355709.1 |
| Mmd2-AD-14 | myotrophin-like |  | XM_027355709.1 |
| Mmd2-AD-12 | myotrophin-like |  | XM_027355709.1 |
| Mmd2-AD-15 | uncharacterized protein |  | XP_037794378.1 |
| Mmd2-AD-1 | myotrophin-like |  | XM_027355709.1 |
| Mmd2-AD-2 | myotrophin-like |  | XM_027355709.1 |
| Mmd2-AD-10 | myotrophin-like |  | XM_027355709.1 |
| Mmd2-AD-23 | myotrophin-like |  | XM_027355709.1 |
| Mmd2-AD-25 | myotrophin-like |  | XM_027355709.1 |

**2 Supplementary Figures**


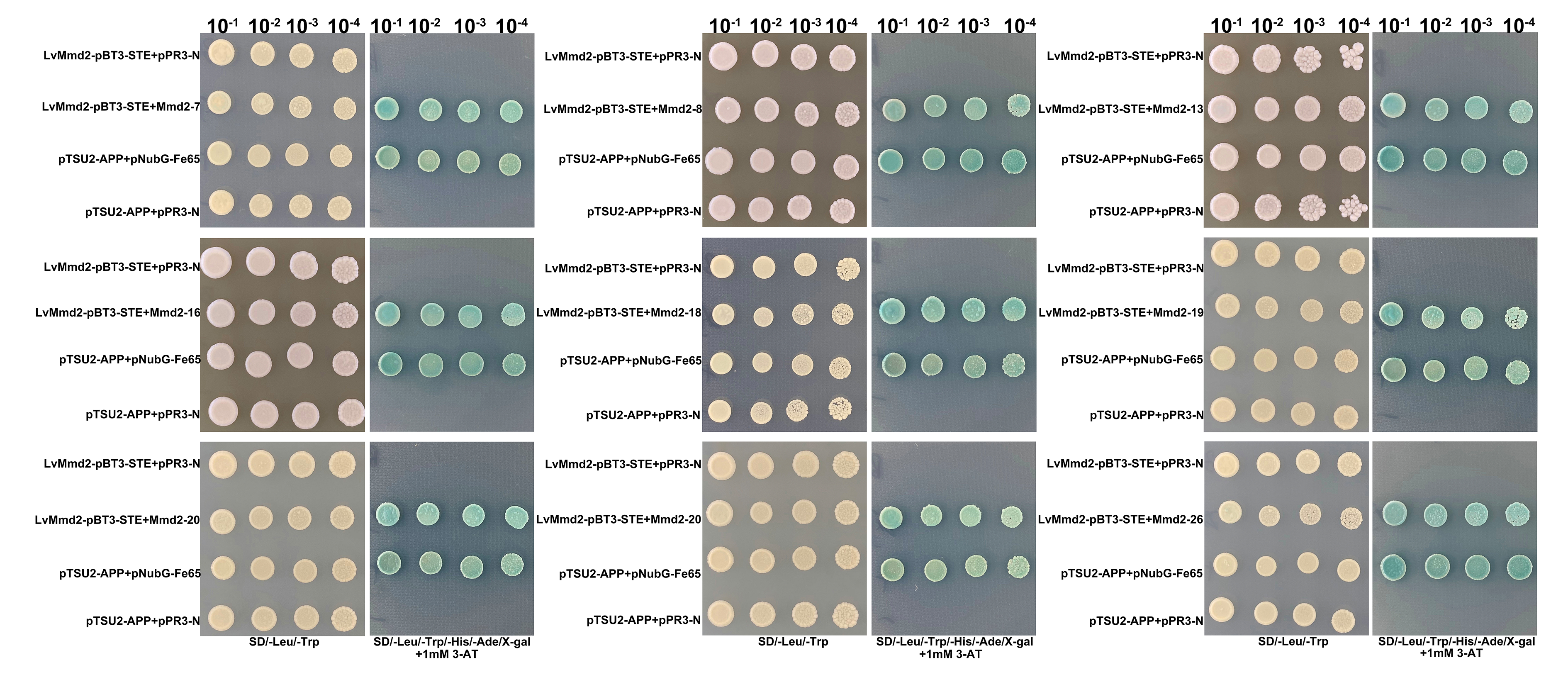


Fig.S1 Support of interaction between 9 positive clones interacting with LvMmd2 by one-to-one yeast two-hybrid.


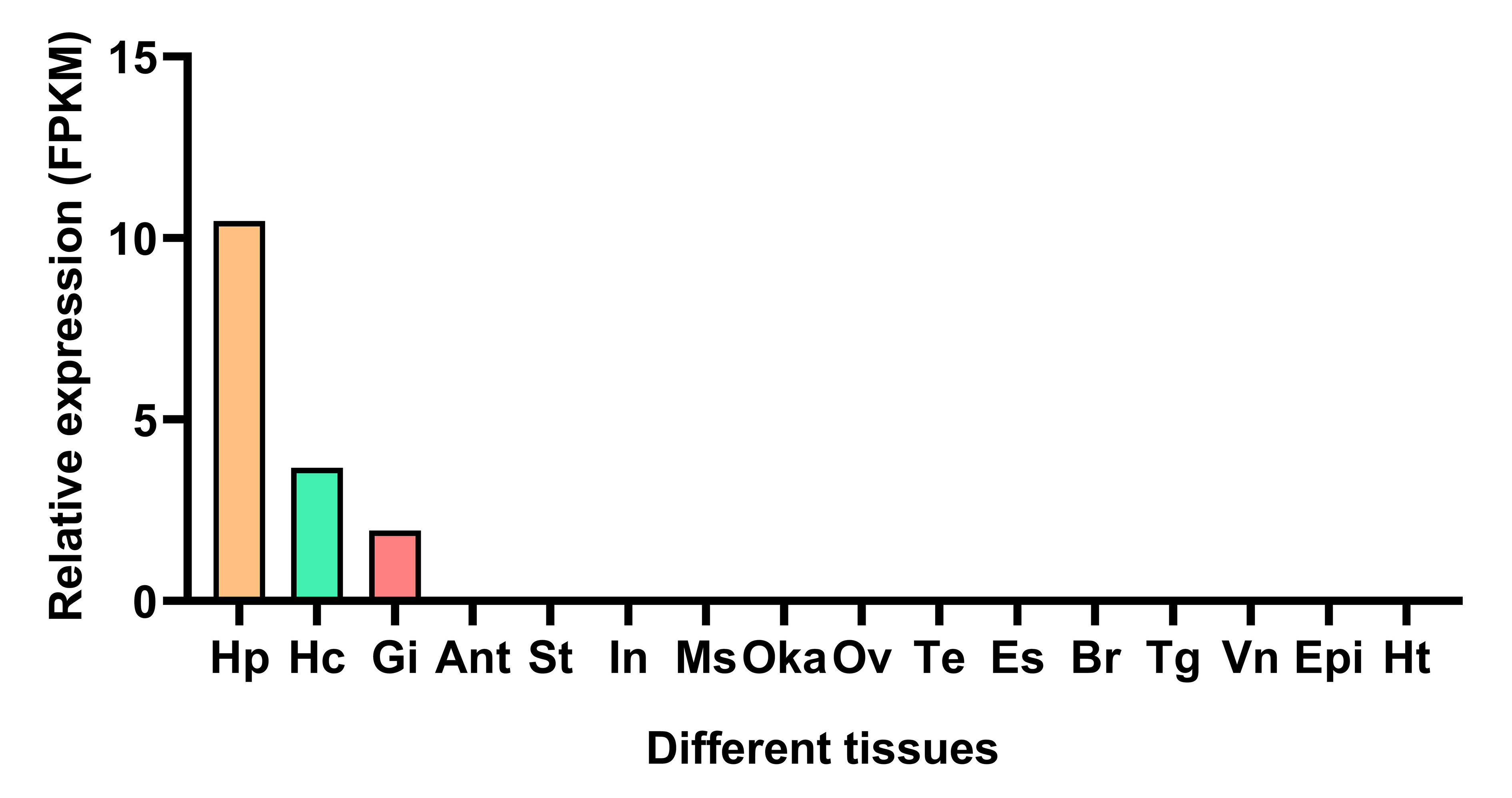


Fig.S2 Tissue distribution of ribonuclease kappa-B-like.


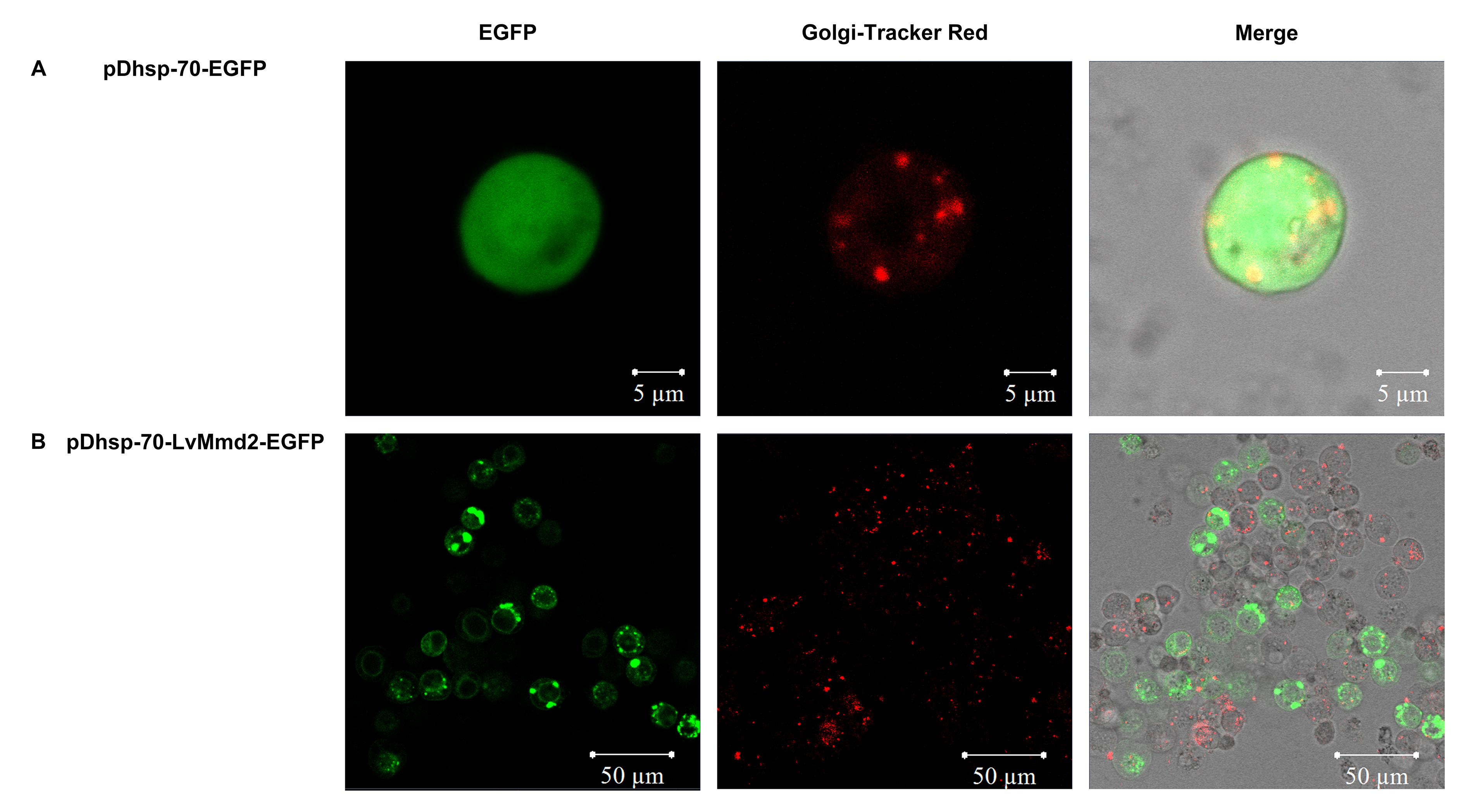


Fig.S3 LvMmd2 may be located on the Golgi apparatus. A EGFP control group is located in the cytoplasm. B LvMmd2 and Golgi markers may be co-located.
